# Comparing the EMS adopter and EMS non-adopter organizations in achieving sustainable business goals

**DOI:** 10.3389/fpsyg.2022.1009457

**Published:** 2022-10-06

**Authors:** Yizhou Wang, Mahmood Rehmani, Muhammad Raees Ashraf, Nazia Farheen, Huda Irshad

**Affiliations:** ^1^School of Humanities, Southeast University, Nanjing, China; ^2^Department of Management Sciences and Economics, Grand Asian University Sialkot, Sialkot, Pakistan; ^3^Department of Business Administration, University of Sialkot, Sialkot, Pakistan

**Keywords:** sustainable development, sustainable business, environmental performance, occupational health and safety performance, employee satisfaction, operational improvement, competitive advantage

## Abstract

The corporate sector has paid attention to the concept of sustainable development since the emergence of this concept in the late 1980s and the adoption of Environmental Management Systems (EMS) by many organizations is the initiative of these organizations’ exact dimensions. The focus of this research is to compare the status of EMS adopter and EMS non-adopter organizations in terms of Environmental Performance, Occupational health and safety performance, Employee Satisfaction, Operational Improvement and competitive advantage. The overall environmental performance of EMS adopter companies was approximately two times higher than non-EMS adopter companies. EMS adopter organizations’ performance was almost two times better than non-EMS adopter organizations regarding occupational health and safety. The overall Employee Satisfaction level at non-EMS adopter organizations was about three times better than at EMS adopter organizations. The EMS adopter organizations were found to have about three times performance regarding operational improvement. Regarding the competitive advantage gained by the EMS adopter companies compared to the non-EMS adopter organizations, no significant difference was observed between these two categories of organizations. However, The EMS adopter organizations seem to have a slight competitive advantage over non-adopter companies.

## Introduction

An environmental management strategy is one in which human activities and their effects on the environment are considered. A detailed description of environmental management has been attempted numerous times. According to Fura (2013), environmental management guides or utilizes various techniques to address environmental and development issues. According to the author, all human activities have a beneficial or harmful impact on the ecosystem. Environmentalists are alarmed by the growing human impact on the environment. Environmental circumstances on which humans depend for survival have been adversely affected by specific processes such as industrialization, urbanization, and an increase in population (Olagunju, 2015). According to Olagunju (2015), pollution is the primary cause of environmental degradation caused by humans. In this approach, environmental management comprises the strategies employed to restrict human impact on the environment. Management of natural resources and other activities is a primary problem for environmental improvement for human survival (Concepts of Environmental Management, 2016). “Quality of life” refers to the basic needs and ethical decisions that should be made when carrying out development operations to meet people’s basic human needs and fulfill their basic human desires. Mental and physical health and wellbeing are the most critical determinants of human wellbeing, and these aspects depend on the degree to which various social contexts meet these criteria (Concepts of Environmental Management, 2016). It became evident that environmental management was required when human activities damaged the quality of human life negatively.

It has become increasingly difficult for businesses to compete with multinational corporations without adopting advanced information and management systems (IMS) such as the Environmental Management System (EMS), which includes some procedures and activities that help companies to reduce their environmental impact and improve their overall performance. One of the most effective EMS examples is ISO 14001, widely regarded as a best practice. Organizations in underdeveloped nations have found it challenging to attain their sustainable development goal because of the costs associated with EMS adoption and implementation.

Organizations must invest significant financial resources in implementing EMS; some of the costs involved are staff training, audits, and auditor fees, as well as EMS certification and its maintenance afterward. Financial Resources: (De Joussineau, 2012). The expense of recording and monitoring critical factors such as air, soil, and water, as well as the cost of surveillance, are extra expenditures associated with EMS; as a result of the considerable expenses associated with EMS, most businesses are hesitant to adopt it. The economic swings, particularly the slowdowns, have harmed the motivation of managers to pursue EMS. Doody (2010) believes that organizations frequently reject the long-term benefits that EMS provides in favor of the short-term profits that can be obtained by not deploying the EMS in the first place.

The International Organization of Standardization created a set of standards for environmental management systems (EMS) that are to be followed worldwide to standardize efforts to improve environmental performance and redirect such actions in the proper direction. It was the ISO 9000 series that became the first series to be formed in 1987 to encourage the use of quality assurance management systems in enterprises (Martincic, 1997). Alternatively, the ISO 14000 standard aids organizations in the management of environmental impacts associated with their business operations (Hemenway and Hale, 1995). ISO 14000 series is being followed at the moment, with ISO 14001 being the most recently updated edition (Martincic, 1997). According to Deepak et al. (2015), the ISO 14000 Series is not a standard in and of itself but rather a collection of environmental management systems (EMS) that address a wide range of environmental-related concerns. It has already been stated that the primary goal of the ISO 14000 series is to assist enterprises in managing and controlling the environmental effects of their operations. Organizations can do this by implementing the ISO 14000 standard, which helps them develop management systems that reduce or eliminate the negative environmental impact of their activities (Hemenway and Hale, 1995). ISO 14000 also ensures that the organization complies with all applicable legal and other necessary environmental obligations, reducing the likelihood of legal liability for the organization. Additionally, it enables enterprises to assess and improve their environmental performance over time (Hemenway and Hale, 1995).

One of the most notable benefits of ISO 14000 is that it substantially contributes to a more robust sustainable development (Hemenway and Hale, 1995). The ISO 14000 standard impacts the design and manufacturing of products, the selection of raw materials, the type of data collected, and how it is transferred internally and externally. It covers the operations, activities, products, services, facilities, and transportation of the company’s products and services (Hemenway and Hale, 1995; Iftikhar et al., 2021). A system of worldwide standards makes it easier to concentrate on the environment and to build a cleaner, healthier, and safer society by encouraging people to do more with less. It also establishes internationally recognized standards against which organizations can measure their progress in environmental protection.

According to Mulyanto et al. (2018), employee performance is the key to productivity because performance results from the human resources and other resources bring the outcome based on the level of quality and standards that have been set. Consequently, organizations need human resources with expertise and unique capabilities following the vision and mission of the organizations. Their study aimed to analyze the effect of competence and environmental management systems on employee performance.

Using an event study, Li and Wu (2017) studied the changes in firms’ performance in profitability, sales, and operational efficiency after environmental management system (EMS) adoption. Based on 22 events of EMS adoption, they found a significant decrease in firms’ profitability, sales, and inventory productivity. They explored the reasons which led to the decline in firm performances. It was found that the increase in sample firms’ total assets was the primary reason. The loss in operational efficiency and flexibility was due to the requirements of the EMS.

Companies are reengineering their processes to be greener and cleaner as customers and society become more conscious of the need for alternative energy sources and environmental sustainability in manufacturing (Islam et al., 2021). Environmental management systems are an example of a best practice that is becoming more widely used. It is important to note that environmental management systems refer to the policies and objectives of an organization as well as the information networks and information management systems used to monitor and evaluate an organization’s environmental impact. It is common to refer to environmental management systems as “green management” systems. ISO 14000 is one of many environmental management systems (EMS) that companies use to ensure they comply with environmental regulations, reduce the risks associated with non-compliance, and cut their environmental costs. For example, ISO/TC207 (2013) explains how these systems aid in the creation of sustainable systems and the training of staff. Each operation’s environmental impact and the company’s performance can be tracked using a formal environmental policy that has been thoroughly documented.

Specific unique characteristics set an Environmental Management System apart from other data and procedure management programs, which businesses commonly use to cut costs and streamline operations. These aims are paired with methods for adhering to strict environmental standards and deadlines in an emergency management system (EMS). The system’s goals and the responsibilities of each stakeholder are outlined in detailed training plans sent to all participants. Detailed records of all the goals, strategies, and timetables have been kept. Everyone involved in achieving the goal or carrying out the process will be given a particular set of responsibilities and a crystal-clear reporting structure. As part of the plan, recommendations for contingency management and a list of corrective measures are provided for each target. Regular audits are essential to ensure that goals are being reached and audit schedules are planned. Performance metrics are created to establish whether the actual performance is in line with the estimated values. Because of this, the EMS can help set attainable objectives, keep tabs on progress, and facilitate ongoing planning.

An environmental management system (EMS) ensures that environmental commitment is integrated into all elements of a company’s operations and that all stakeholders know their long-term sustainability responsibilities (Rehmani and Khokhar, 2018). As a result, the company’s reputation and trustworthiness are enhanced. As indicated by the unexpected rise in demand for organic items and eco-friendly autos in developing economies, consumers are becoming more environmentally conscious. Sustainability activities can positively impact the financial health of an organization as a result of environmental awareness and commercial support.

EMS, aside from standard environmental management systems like EMAS and EMS, businesses are increasingly focused on environmental sustainability systems that include environmental accounting, reporting, and life cycle assessment (LCA). These strategies aim to make firms more environmentally friendly by increasing their environmental commitment. In addition to reducing the company’s carbon footprint, an EMS adds to greater transparency and accountability by ensuring that procedures and regulations are correctly documented.

ISO 14000 certified companies in Greece have gained a better position in their market, transitioned from environmentally damaging practices to environmentally sustainable ones, and have better relationships with society because of their superior environmental performance, according to an investigation of 53 of these companies (Psomas et al., 2012). Research has shown that companies with environmental management systems (EMS) have a competitive advantage over those without EMS systems as stakeholders become more aware of environmental degradation.

When the environmental management system (EMS) is not followed, the safety and health of workers on the job are also jeopardized, which indirectly impacts the organization’s performance (Daily et al., 2011). When employees become aware that adequate measures to ensure their safety have not been taken, they become distracted by their concern for their safety and consequently do not perform as well as they can.

The image businesses present is increasingly vital in today’s world; the public perceives organizations implementing EMS as better corporate citizens concerned about the health and betterment of the community. Consumers are also more likely to purchase from companies that practice environmentally friendly business practices. Governments can encourage the use of EMS by offering tax breaks to those who do so. If a firm does not adhere to EMS, the organization’s value to its shareholders also decreases.

When the considerations mentioned above are weighed against one another, it becomes clear that implementing an EMS is good for the environment and the organization itself. There is a gap in the existing literature regarding the correlation between environmental performance, occupational health and safety, operational improvement, employee satisfaction and competitive advantage. It is a valuable technique for lowering overhead costs, allowing for more efficiency. In addition, it should be highlighted that the literature focuses solely on the benefits provided by EMS and the advantages offered by the various EMS models available. There have been a few studies done, and they have identified multiple reasons for not implementing EMS, with an emphasis on costs and employee resistance. This study will focus on studying the status of the variable mentioned above in EMS adopter and non-adopter firms besides the correlation among all these variables.

The environmental strategy is designed to assist organizations in improving performance levels and gaining a competitive edge. It also serves as a tool to enhance income while decreasing costs (Baroto et al., 2012). The increase in revenues and the decrease in expenses specify environmental management strategies that call into question the optimism of the situation (Baroto et al., 2012). The environmental advocates for optimism assist in involving optimism to incorporate projects and practical environmental experience in the environmental movement (Alexander and Poyyamoli, 2014). Using the environmental management statements for Emerald and Science Direct, we may investigate the justifications made in support of the ISO 14000 and ISO14001 standards, respectively.

To indicate good green promotional benefits due on sales, those benefits of being ahead of competitors must be communicated. Greater returns on capital investment for improvement in green investment present an opportunity for early initiatives for business improvement to be implemented. Improved environmental conditions for selection effects to pursue accessible money increase the profitability of businesses.

It has been demonstrated by Watson et al. (2004) that organizational performance may be quantified for adopter and non-adopter of environmental management systems (EMS). Monitoring, managing, and improving performance linked with the organization’s internal and external environments are all made possible through environmental management standards (EMS). Compliance with environmental management systems (EMS) and ISO standards improves the efficiency of an organization. According to the findings, the association between EMS implementation and Environmental Performance received mixed assessments. A statistically significant relationship between implementing an EMS and organizational and managerial efficiency by Biondi et al. (2000).

On the other hand, Sambasivan and Fei (2008) highlighted several benefits of EMS deployment, including increased employee motivation and performance, greater profitability, and increased customer loyalty. Furthermore, adopting an EMS improves corporate control, lowers environmental accidents, promotes staff motivation, and improves the operational efficiency of an organization. The company’s size also determines the extent to which the implementation of an EMS will provide operational and financial benefits. The deployment of an enterprise resource planning system (ERP) significantly impacts operational and corporate performance by expanding their capabilities.

This study was conducted on the EMS adopter and non-EMS adopter companies of Gujranwala, Sialkot, Lahore, Gujrat, and Faisalabad districts of Pakistan. This study portrayed a picture of the EMS adopter industries and the non-EMS adopter companies, which are supposed to comply with government regulations. It may be noted that the EMS non-adopter industries also undergo the process of Environmental Impact Assessment, devising of a mitigation plan for coping Environmental, social and economic impacts which further leads to designing of a formal Environmental Management plan including continuous monitoring and check and balance by the entailing government agencies. In case of effective implementation and monitoring of EMP, there is supposed to be good performance regarding environmental, social and economic variables.

The research objectives of this study include the following:

1. Is there significant difference between the Environmental performance of EMS adopter and non-EMS adopter companies besides evaluation of CSR performance at both categories of organizations?
2. Is there significant difference between the occupational health and safety performance of EMS adopter and non-EMS adopter companies besides evaluation of CSR performance at both categories of organizations?
3. Is there significant difference between the employee satisfaction level of EMS adopter and non-EMS adopter companies besides evaluation of CSR performance at both categories of organizations?
4. Is there significant difference between the operational performance of EMS adopter and non-EMS adopter companies besides evaluation of CSR performance at both categories of organizations?
5. Is there significant difference between competitive advantage of EMS adopter and non-EMS adopter companies besides evaluation of CSR performance at both categories of organizations?

## Methodology

**Figure 1:**
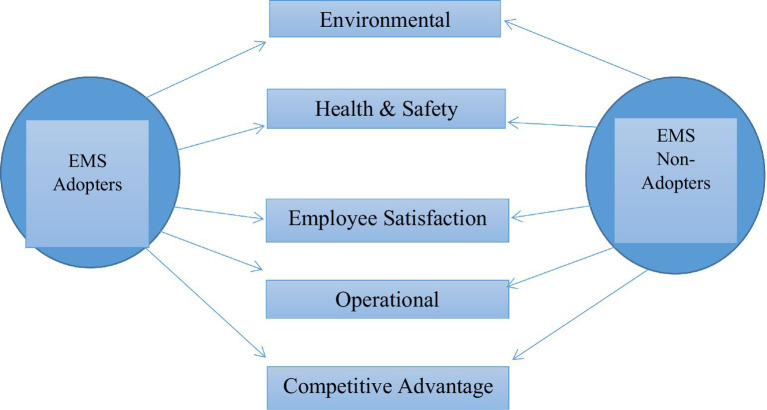
Theoretical framework.

The performance of EMS adopter and non-EMS adopter companies (independent variables) will be evaluated through dependent variables, which include Environmental Performance, Health and Safety, Employee Satisfaction, Operational Improvement, and Competitive Advantage. The model is shown in the following section as Figure 1.

This study focused on organizations that adopted environmental management systems and those that did not. The questionnaire based on the Likert scale was used to collect data, and R language and MS Excel were used to analyze the results. Study objectives, ethics and privacy of information were explained to respondents. Data was gathered through the distribution of questionnaires in 30 organizations, selected based on 15 EMS companies and 15 non-EMS companies. The sampling design was a two-stage simple random sampling utilized by the following procedure.

Gujrat, Faisalabad and Gujranwala do not have many organizations, so we chose one EMS and non-EMS companies from each city. On the other hand, Sialkot and Lahore have more organizations, so we decided on six EMS and six non-EMS companies from both cities. In total, 24 companies belonged to Sialkot and Lahore while the rest were from Gujrat, Faisalabad and Gujranwala. A sample of 10 respondents was selected from each company by simple random sampling. So, a total of 300 samples were collected and after data cleaning, the remaining final sample size was 290 respondents.

The statistical analysis for this study includes Descriptive Statistics, Exploratory Factor Analysis (EFA), Reliability Analysis, Correlation analysis and logistic regression analysis. We choose the logistic regression analysis to determine the relationship between OI, CA, ES, HS, and EP. It is used to predict the chances of an event between two scenarios such as EMS and non-EMS adopter companies affecting performance.

## Analysis and results

Table 1 shows that most factor loading is above 0.60, whereas some are above 0.50 and only two items have factor loading below 0.5 but above 0.4, which shows no need to exclude any additional items.

**Table 1:** Factor loadings of principal component analysis.

Question	Question statement	F. loading
OI1	The adoption of EMS at your organization has reduced the number of accidents at workplace.	0.61
OI2	The adoption of EMS has resulted in efficient use of raw materials/ or any other inputs.	0.60
OI3	The adoption of EMS has resulted in energy efficiency at your organization.	0.61
OI4	The adoption of EMS has resulted in less waste generation.	0.68
OI5	The adoption of EMS has resulted in less emissions.	0.68
OI6	The adoption of EMS has resulted in less noise.	0.64
OI7	The adoption of EMS has resulted in lower operational cost.	0.62
OI8	The adoption of EMS has resulted in higher production.	0.54
CA1	Your company enjoys good level of customer loyalty.	0.69
CA2	Your company enjoys a good image which is improving with time.	0.68
CA3	Your company uses environmental slogans or green labeling.	0.66
CA4	Your company uses recycled materials for manufacturing or other purposes.	0.55
CA5	Your company uses recyclable materials for manufacturing or other purposes.	0.79
CA6	Your company spends on environmental restoration activities or initiatives.	0.82
EP_P1	Your organization has identified its major environmental issues.	0.53
EP_P2	Your organization has established procedures to identify the organizational environmental aspects.	0.70
EP_P3	The aspects having significant impacts on the environment have been determined.	0.66
EP_P4	The legal requirements related to your organization have been identified.	0.70
EP_P5	Your organization has established Environmental objectives and targets.	0.68
EP_P6	Environmental management program has been devised to achieve environmental objectives and targets.	0.74
EP_P7	The rules and regulations have been given to staff for achieving environmental objective and targets.	0.65
EP_P8	Each environmental objective and target has been quantified where practicable.	0.73
EP_P9	Environmental objectives have been set with a relevant timeframe.	0.61
EP_P10	The critical operations / staff who may create more hazard, been identified	0.57
EP_P11	The organization has procedure to respond to accidents and emergency situations?	0.62
EP_P12	New developments/ modified activities, products or services have been incorporated in EMP.	0.60
EP_IO1	The roles and responsibilities are well defined.	0.46
EP_IO2	The environmental management system is beneficial.	0.64
EP_IO3	Financial resources are made available to implement environmental management program.	0.70
EP_IO4	A procedure exists to respond to accidents and emergency situations.	0.70
EP_IO5	Enough human resources and specialized skills, technology, resources are available.	0.69
EP_IO6	All personnel whose work may create a significant impact upon the environment receive appropriate training.	0.81
EP_IO7	The organization maintains procedures for Internal communication between the various levels and functions.	0.69
EP_IO8	The external interested parties are coordinated on environmental aspects for effective EMS implementation.	0.69
EP_IO9	The contents of training are focused on significant impacts upon the environment.	0.76
ES1	There is adequate noise control to allow me to focus on my work.	0.39
ES2	I feel physically safe in my work environment.	0.57
ES3	This organization provides as much initial training on environment, health and safety as I need.	0.80
ES4	This organization provides as much ongoing training on health safety and environment as I need.	0.69
ES5	This organization encourages me to develop professionally and/or acquire new skills	0.70
ES6	My organization provides good working environment.	0.63
HS1	The procedure exists to measure risk level of critical operations (that may cause significant environmental impacts).	0.57
HS2	The organization has assigned the responsibility for handling and investigating non-conformances.	0.64
HS3	The procedure for checking and corrective action includes the recording of information to monitor performance.	0.70
HS4	EMS audit is carried out periodically.	0.70
HS5	The organization has established and maintained procedures for mitigating possible impacts.	0.59
HS6	EMS audit records are kept for future references and continual improvement.	0.64
HS7	The causes of actual and potential non-conformances are eliminated through checking and corrective action.	0.63

**Figure 2:**
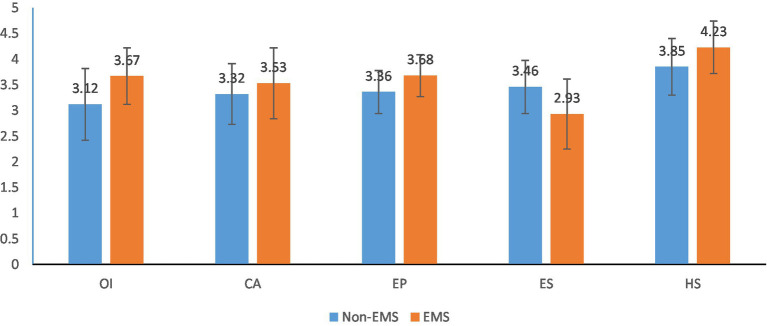
Comparison of the performance of EMS and non-EMS.

Table 2 shows that all the items are well classified as each item is placed in its factor. At the same time, the six factors are sufficient for the item’s classifications (Table 3). Table 4 and Figure 2 show that Operational Improvement, Competitive Advantage, Environmental Performance and Health and Safety has a comparatively high average value in EMS Companies than the non-EMS companies. However, the Employees Satisfaction is reverse here, and Employees Satisfaction is more in non-EMS companies than in EMS companies.

**Table 2:** Factor classification.

Items	Factor 1	Factor 2	Factor 3	Factor 4	Factor 5	Factor 6
EP_P1	0.67					
EP_P2	0.51					
EP_P3	0.56					
EP_P4	0.51					
EP_P5	0.51					
EP_P6	0.47					
EP_P7	0.56					
EP_P8	0.5					
EP_P9	0.6					
EP_P10	0.63					
EP_P11	0.56					
EP_P12	0.61					
OI1		0.57				
OI2		0.58				
OI3		0.6				
OI4		0.5				
OI5		0.49				
OI6		0.58				
OI7		0.58				
OI8		0.61				
EP_IO1			0.71			
EP_IO2			0.57			
EP_IO3			0.52			
EP_IO4			0.51			
EP_IO5			0.55			
EP_IO6			0.4			
EP_IO7			0.53			
EP_IO8			0.53			
EP_IO9			0.48			
HS1				0.54		
HS2				0.52		
HS3				0.43		
HS4				0.51		
HS5				0.58		
HS6				0.54		
HS7				0.55		
ES1					0.73	
ES2					0.63	
ES3					0.41	
ES4					0.53	
ES5					0.53	
ES6					0.6	
CA1						0.55
CA2						0.55
CA3						0.57
CA4						0.66
CA5						0.44
CA6						0.41

Chi-Square 950.38. *p*-value 0.0125.

**Table 3:** Reliability statistics.

Variable	No. of items	Cronbach’s alpha
Operational improvement	8	0.817
Competitive advantage	6	0.699
Environmental performance planning	12	0.858
Environmental performance implementation and operation	9	0.784
Environmental performance	21	0.799
Employees satisfaction	6	0.754
Health and safety	8	0.69

The Cronbach’s Alpha value is almost 0.70 and higher, which indicates that our collected data is reliable and internally consistent.

**Table 4:** Descriptive data analysis.

Company		Min.	1st quartile	Median	Mean	3rd quartile	Max.
Non-EMS	OI	1.38	2.630	3.130	3.116	3.630	4.750
	CA	1.670	2.830	3.415	3.324	3.670	4.500
	EP	2.340	3.070	3.430	3.363	3.660	4.200
	ES	2.330	3.000	3.330	3.463	3.830	4.670
	HS	1.570	3.570	3.710	3.849	4.178	5.000
EMS	OI	1.880	3.500	3.750	3.673	4.000	4.750
	CA	1.67	2.83	3.75	3.53	4.00	4.83
	EP	2.450	3.455	3.750	3.680	3.970	4.530
	ES	1.330	2.500	3.170	2.929	3.500	4.000
	HS	1.710	4.140	4.430	4.234	4.570	5.000

**Figure 3:**
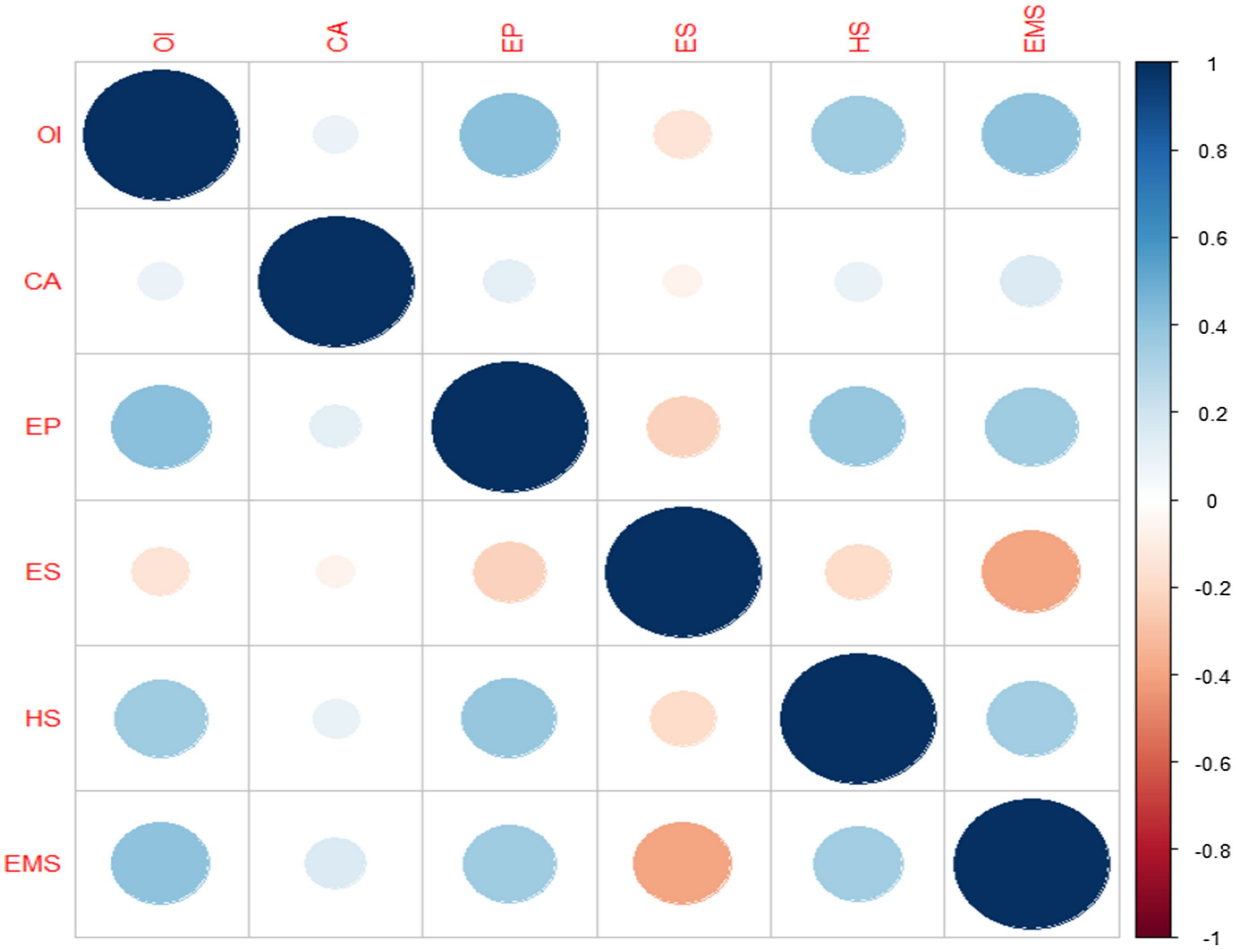
Correlation analysis.

Figure 3 shows that EMS is moderately correlated with Operational Improvement, Environmental Performance, Health and Safety whereas moderately negatively correlated with Employee satisfaction and very mildly correlated with Competitive Advantage.

Environmental Performance, Health and Safety and Operational Improvement are also moderately correlated. In contrast, all other pairs are mildly correlated except Employees Satisfaction which is mildly associated with other variables (Table 5).

**Table 5:** Correlation matrix.

Variables	OI	CA	EP	ES	HS	EMS
OI	1					
CA	0.088	1				
EP	0.411^***^	0.115	1			
ES	−0.145^*^	−0.065	−0.228^***^	1		
HS	0.359^***^	0.096	0.380^***^	−0.186^**^	1	
EMS	0.4058^***^	0.157^**^	0.356^***^	−0.398^***^	0.341^***^	1

* signficant at 0.05 ** significant at 0.01 *** significant at 0.001.

### Regression analysis

Table 6 shows that the Operational Improvement, Environmental Performance, Employees Satisfaction and Health and safety are all statistically significant. In contrast, only one variable Competitive Advantage is non-significant with a *p*-value of 0.065. The Chi-square value of deviance difference is 113.97 with DF 5, and the associated *p*-value is 0.000. This tells us that our model fits significantly better than an empty model. Logistic regression is used to measure the probability of an event, so we use odds ratio values to predict the impact of variables (Sperandei, 2014). It is useful as we study whether EMS are better than non-EMS companies.

**Table 6:** Coefficients estimate.

Variable	Estimate	SE	z-value	Pr(>|z|)	Odds ratio	2.5%	97.5%
(Intercept)	−5.8444	1.8218	−3.208	0.001^**^	0.002	7.41E − 05	0.096289
OI	1.0548	0.2528	4.173	0.000^***^	2.871	1.77E + 00	4.794524
CA	0.4076	0.221	1.844	0.065	1.503	9.79E − 01	2.333519
EP	0.7751	0.3755	2.064	0.039^*^	2.171	1.05E + 00	4.586686
ES	−1.4205	0.2654	−5.351	0.000^***^	0.242	1.40E − 01	0.397811
HS	0.6845	0.2949	2.321	0.020^*^	1.983	1.13E + 00	3.599366

* signficant at 0.05 ** significant at 0.01 *** significant at 0.001.

The Odds Ratio of Operational Improvement is 2.87, meaning that the likelihood of Operational Improvement is about three times higher in EMS companies than in non-EMS companies.

The Odds Ratio of Competitive Advantage is 1.5 which means that the likelihood of Competitive Advantage is about one and half times higher in EMS companies than in non-EMS companies. However, this is statistically non-significant.

The Odds Ratio of the likelihood of Environment Performance is 2.17, meaning that Environment Performance is about two times higher in EMS companies than in non-EMS companies.

The Odds Ratio of Employee Satisfaction is 0.24, meaning that the likelihood of Employee Satisfaction is about two times lower in EMS companies than in non-EMS companies. We can also say 76% less satisfaction in EMS than in non-EMS.

The Odds Ratio of Health and Safety is 1.98, meaning that the likelihood of Health and Safety is about two times higher in EMS companies than in non-EMS companies.

## Discussion

### Environment performance

The overall environmental performance of EMS adopter companies was approximately twice as high as that of non-EMS adopter companies. Our results are in line with the findings of Psomas et al. (2012), who stated that ISO 14000 certified companies in Greece had gained a better position in the market. They transitioned from environmentally damaging practices to environmentally sustainable ones and had better relationships with society because of their superior environmental performance. Li et al. (2019) studied a sample of 100 listed companies in China. They found that EMS has a significant positive relationship with corporate green innovation and that environmental regulation strengthens this link. EMS may enhance efficiency by improving waste management processes (Zutshi and Sohal, 2005).

#### Planning

It was found that the EMS adopter companies identify their environmental issues more efficiently than the non-EMS adopter. The procedures are established more candidly by the adopter organizations. The adopter determine the environmental aspects as a legal obligation. The adopter organization’s objectives and targets are set more efficiently to achieve environmental performance. The environmental management programs at adopter organizations are also devised accordingly.

Regarding environmental compliance by the relevant staff, the rules and regulations are communicated more formally than in non-adopter organizations. To measure the results or performance of environmental objectives at adopter organizations, the same are quantified in measurable form. Adopter organizations also set a justified time frame for achieving the environmental objectives. The critical operations that may cause hazards at EMS adopter organizations were also identified. The EMS adopter organizations were found to have more efficient procedures for responding to accidents and emergencies. The EMS adopter organization was also found more innovative in the case of modified activities, products, or services regarding their incorporation in environmental management programs.

#### Operational implementation

EMS adopter organizations well defined the rules and responsibilities. The financial resources, human resources, specialized skills, and technologies were made available to implement environmental management programs at adopter organizations. The training requirement was also exploded more efficiently by the adopter companies. More sound procedures existed at adopter companies to respond to accidents and emergencies. The personnel at adopter organizations received appropriate training to cope with environmental issues. There was honest communication at adopter organizations regarding environmental problems and hazards, leading to good coordination regarding the implementation of EMS. Fura (2013) also narrated that an environmental management system (EMS) is a helpful tool that helps companies improve their energy efficiency and environmental sustainability by designing processes and procedures to govern environmental impact. Fei-Baffoe et al. (2013) also stated that in a study on the implementation of environmental management systems (EMS) in gold mining industries, the adoption of EMS resulted in significant improvements in environmental performance, including better waste management, more efficient use of energy resources, and a reduction in incidents at the site.

### Health and safety

EMS adopter organizations’ performance was almost twice as good as non-EMS adopter organizations regarding occupational safety. At EMS adopter organizations, the sound procedure existed to measure the risk level of critical operations. These organizations were assigned to handling and investigating non-conformances in this regard. The health and safety performance were measured efficiently, and corrective actions were taken at adopter organizations. The EMS audits were carried out periodically at adopter organizations which were the co-reason for better health and safety performance. The EMS adopter organizations maintained a log of incidents and impacts which was found lacking at most non-adopter organizations. EMS audits records maintained by adopter organizations served as a good source for continual improvement of health and safety practices. The corrective actions and heading of due attention to non-conformances at EMS adopter organizations lead to sound environmental, health, and safety performance. Our results are in line with the findings of Daily et al. (2011). They state that when the environmental management system (EMS) is not followed, the safety and health of workers on the job are also jeopardized, which indirectly impacts the organization’s performance. They further narrated that when employees become aware that adequate measures to ensure their safety have not been taken, they become distracted by their concern for their safety and consequently do not perform as well as they could. The public perceives the organizations implementing EMS as better corporate citizens concerned about the community’s health and betterment. Consumers are also more likely to purchase from companies that practice environmentally friendly business practices. Zaman et al. (2022), Daily et al. (2011) stated that EMS implementation functioned as a source of difference from competitors and a means of reducing employee health and safety hazards.

### Employee satisfaction

The overall Employee Satisfaction level at non-EMS adopter organizations was about three times better than at EMS. Pojasek (2012) also states that by enhancing the company’s reputation and image, raising employee morale and motivation, and boosting earnings, EMS has a good effect on the organization’s overall performance, as well as increasing consumer trust and loyalty. Our findings are in line with Ikram et al. (2019), who furnished that Non-EMS companies had a positive and substantial impact on employee satisfaction and economic contribution compared to EMS companies. The non-EMS adopter organizations employees exhibited more complacency regarding their workplace environment. The issue of noise was not considered a calamity by the workers at non-EMS adopter organizations. Similarly, these workers generally faced no physical safety issues at their organizations. The employees at non-EMS adopter organizations did not consider training on environment, health, and safety as essential to rate their organizations as safe. Furthermore, acquiring new skills and professional developments were not found to be the priority areas of the workers at non-EMS adopter organizations. The logical reason for higher employee satisfaction at non-EMS adopter organizations seems to be a lack of awareness and immediate financial and social issues. Especially the vicious circle of poverty, non-implementation or non-willingness of the employers to implement health and safety policies in the best interest of the employees, and the need to go through training programs, which is considered a futile practice by mostly illiterate workers. And finally, there is the ineffective implementation of the rules and regulations by the government’s institutions which also affects employee satisfaction.

### Operational improvement

The EMS adopter organizations were found to have about three times better performance. The number of accidents was reduced at EMS adopter organizations. The raw materials were found to be used more efficiently at these originations. Regarding energy efficiency, the EMS adopter organizations were again the better performance. The EMS adopter organizations also produced less waste and emissions. The issue of noise pollution was also found to be less severe at The EMS adopter organizations compared to The Non-EMS adopter. It was found that the adoption of EMS resulted in higher operational costs in the beginning but resulted in higher economic gains in the long term. It was further found that the production of EMS adopter organizations increased over time, reckoning toward the image improvement and ultimate marketing gains to the EMS adopter. Daughtry (2014) believes new targets and objectives should be introduced to replace those completed in the past to achieve continuous improvement. Our results are supported by who investigated the influence of the ISO 14001 Environmental Management System (EMS) on the fashion and textile industries and discovered some encouraging results. In the author’s opinion, the operations of the textile industry generate large amounts of waste, which was previously dumped in rivers, resulting in water pollution (Rehmani et al., 2022). However, the implementation of ISO 14001 resulted in a reduction in wastage, as well as the proper and systematic disposal of waste through recycling, efficient work procedures, scheduling, and periodic audits. The industry has not only improved its environmental performance but has also improved its economic efficiency. According to Christini et al. (2004), the positive impact of the EMS on the construction industry was visible in the reduction of waste and the improvement of energy efficiency, both of which resulted in cost savings. Low et al. (2015) also reported increased operational efficiency and cost savings, as well as increased employee motivation which is in line with the findings of this study.

### Competitive advantage

Regarding the competitive advantage gained by the EMS adopter companies compared to the non-EMS adopter organizations, a significant difference was not observed between these two categories of organizations. However, The EMS adopter organizations seem to have a slight competitive advantage over non-adopter. While discussing customer loyalty, the EMS and non-EMS adopter organizations enjoyed an excellent level irrespective of EMS adoption or no adoption factors. Many EMS non-adopter companies wanted a good brand image which seems to be due to their product or service quality instead of the EMS adoption factor. Although many EMS adopter companies used environmental or green-making tools, they did not seem to surpass the non-adopter companies to an appreciable extent, possibly due to the low concern level of consumer-customers regarding environmental issues. The priority of the non-EMS adopter companies was neither found to be the use of recycled materials for manufacturing purposes nor its seem to give any competitive advantages to the EMS adopter organizations. The EMS adopter organizations were neither found spending on environmental restoration activities nor intended to. The perception of the non-EMS adopter organizations regarding any profit gains due to environmental initiatives was not good as adopting EMS negatively affected firm performance (Lo et al., 2012). According to Daily et al. (2011), EMS functioned as a source of difference from competitors. Low et al. (2015) investigated environmental management systems in the manufacturing industry. They noticed the benefits that manufacturing firms, particularly export-oriented organizations, reaped. The authors note that one of the most significant benefits of complying with ISO 14001 is that it removes trade obstacles, facilitating international commercial transactions. Compliance with ISO 14001 positioned them as environmentally conscious businesses, and as a result, they were able to secure international contracts.

## Implications of the study in relation to SDGs

The concept of sustainable development came into being because of United Nations efforts in the form of a report of a Commission constituted in 1980s with an intention to bridge the gaps between world’s haves and have nots. The concept of sustainable development was based on the intentions to save the planet earth despite the challenges in the form of rapidly growing human population size and consequent industrialization to cater the needs of growing population. The industrial development is the backbone of a country’s economy, but it also gives rise to environmental pollution. Though industrialization provides employment opportunities to the growing population, yet it also poses challenges in the form of human health and safety. The prime focus of all organizations is to earn profits but emergence of global ethics and requirements in the form of sustainable business concept and sustainable development have made it essential for the organizations to take into consideration the environmental, social, and economic performance. The Environmental Management System and Corporate Social Responsibility are the management tools for organizations to march with the global trends and requirements. The core objectives of these practices or systems are to achieve environmental, operational, occupational health and safety performance besides ensuring the employees satisfaction and competitive advantage. The EMS and CSR are meant to achieve the following sustainable development goals.

Goal 3: Good Health and well Being.
Goal 6: Clean water and sanitation.
Goal 7: Affordable and clean energy.
Goal 8: Decent work and economic growth.
Goal 9: Industry, innovation and infrastructure.
Goal 12: Responsible consumption and production.
Goal 13: Climate action.
Goal 17: Partnerships for the goals.

The above listed pertinent goals are indirectly related to Goal No.1 for elevating the human poverty from the planet, earth which may further lead to zero hunger on this planet. The three dimensions of sustainability, i.e., environment, society and economy lead to the dilated concepts of environmental, social and economic sustainability. The core objectives of EMS and CSR are to make a business sustainable while ensuring all these dimensions of sustainability.

## Limitations

The questions were based on judgment of the respondents and in case of Non EMS adopter organizations, the workers were not able to comprehend the questions regarding Environment and safety and verbal briefing was found necessary in such cases which required more time and effort. The workers were also reluctant to respond to the questions where they thought that the image of the organization may be portrayed negatively and they may face any adverse consequences as a reaction to such response. Regarding the impact and effectiveness of Environmental Management System and Corporate Social Responsibility on the business performance, the respondents answered on the basis of their experience where the mentioned practices had been adopted for considerable time period whereas others responded on the basis of their limited knowledge instead of experience. Furthermore, the role of the government and the regulatory bodies in the understanding, implementation and facilitation of the EMS system was not covered in this research. Information regarding EMS and CSR practices and performance was not freely accessible for evaluation, because the developers did not have an authority to showcase them. Some of them were uncertain to display them due to confidentiality issues. Possibly, due to lack of fairness and transparency especially regarding worker rights and safety facilities, many industries were not willing to allow the researchers to their industries assuming it as a risk to compromise their organization secrets. Hence, some developers could not permit the researcher to conduct the study in their sites as a matter of policy. In some industries, only the managers acknowledged being cross-examined, but never the workers. In many cases the Environment or their own safety was not the top priority of the workers due to vicious poverty circle and their entanglement in the same to strive for the basic necessities of life.

## Future directions related to intersection of green practices and digital technology

Though, the two concepts, i.e., Green Practices (Environmental Sustainability) and Digital Technology apparently seem entirely distinct and irrelevant yet they may reinforce each other owing to the use of advanced form of human developed digital technologies starting with internet/5G technology to Artificial Intelligence (AI). The digital technologies have helped the scientists and the environmentalists to collect the environmental data conveniently and efficiently. Currently, the digital technology and environmental sustainability seem exclusive concepts because the factors that propel them are entirely different. The digital technology refers to technological change with the help of internet/5G technology, Artificial Intelligence and robotics which have the goal to transform global manufacturing, industrial processes and labor. Hence, purpose of digital technology is to improve the efficiencies. On the other hand, the green practices (Environmental Sustainability) are based on the concepts of climate change and environmental degradation, which further emphasis on resource conservation, environmental governance and DE carbonization of atmosphere. Owing to rapid increase in population size and consequent linear increase in production and consumption, innovation is required in business to address the ecological and social issues of the day. The future innovative approach would require the mutual reinforcement of the concepts of Environmental Sustainability and Digital Technology. It means that the companies will need to depend on digital technology to ease their pollution foot-prints and manage wastes. In the same way, the energy drawn by computers and other devices can be wasted without understanding the concept of sustainability. Hence, taking these both concepts into an integrated form, the companies may gain long term viability among customers, regulators and the communities where businesses operate. The use of digital technologies with the aim of achieving business sustainability through effective product life cycle assessment may secure the products from being converted into the waste by increasing the overall life span of the products, hence, leading to the concept of circular economy.

## Conclusion

This research article focuses on evaluating the overall performance of the corporate sector to achieve the pertinent sustainable development goals through adopting Environmental Management Systems (EMS; Smorodinova and Cowie, 2002). Apropos, this study compares the status of EMS adopter and EMS non-adopter organizations in terms of Environmental Performance, Occupational health and safety performance, Employee Satisfaction, Operational Improvement, and competitive advantage.

EMS adopter companies’ overall environmental performance was approximately twice better than of non-EMS adopter companies. The EMS audits were carried out periodically at adopter organizations. The EMS adopter organizations maintained a log of incidents and impacts, so they were found to have more efficient procedures for responding to accidents and emergencies. Appropriately corrective actions lead to sound environmental, health, and safety performance. The EMS adopter organizations well defined the roles and responsibilities. The financial resources, human resources, specialized skills, and technologies were available to implement environmental management programs at adopter organizations. The personnel at adopter organizations received appropriate training to cope with environmental issues.

The overall Employee Satisfaction level at non-EMS adopter organizations was about three times better than at EMS adopter organizations. The EMS adopter organizations were found to have much better operational improvement performance. The raw materials were found to be used more efficiently at these originations. Regarding energy efficiency, the EMS adopter organizations were again the better performers. The EMS adopter organizations also produced less waste and emissions. The issue of noise pollution was also found to be less severe at the EMS adopter organizations compared to the non-EMS adopter. It was further found that the production of EMS adopter organizations increased over time, reckoning toward the image improvement and ultimate marketing gains to the EMS adopter.

Regarding the competitive advantage gained by the EMS adopter companies compared to the non-EMS adopter organizations, a significant difference was not observed between these two categories of organizations. While talking about customer loyalty, both The EMS adopter and non-EMS adopter organizations enjoyed an excellent level irrespective of EMS adoption or no adoption factors. Although many EMS adopter companies used environmental or green-making tools, they did not seem to surpass the non-adopter companies to an appreciable extent possible due to the low concern level of consumers/customers regarding environmental issues. The priority of the non-EMS adopter companies was neither found to be the use of recycled materials for manufacturing purposes nor it seem to give any competitive advantages to the EMS adopter organizations. The EMS adopter organizations were neither found spending on environmental restoration activities nor do they seem to intend so. The non-EMS adopter organizations’ perception of any profit gains due to environmental initiatives was also pessimistic. Conclusively, the EMS adopter companies surpassed the non-EMS adopter companies in a cumulative perspective.

## Data availability statement

The raw data supporting the conclusions of this article will be made available by the authors, without undue reservation.

## Author contributions

MR and MA contributed to the conception and design of the study, while NF improved the draft sections. YW refined and improved the manuscript. HI prepared the manuscript for submission. All authors contributed to manuscript revision, read, and approved the submitted version.

## Funding

This work was partly sponsored by the National Social Science Foundation of China (19ZDA040).

## Conflict of interest

The authors declare that the research was conducted in the absence of any commercial or financial relationships that could be construed as a potential conflict of interest.

## Publisher’s note

All claims expressed in this article are solely those of the authors and do not necessarily represent those of their affiliated organizations, or those of the publisher, the editors and the reviewers. Any product that may be evaluated in this article, or claim that may be made by its manufacturer, is not guaranteed or endorsed by the publisher.
